# Sternal intraosseous schwannoma mimicking breast cancer metastasis

**DOI:** 10.1186/1749-8090-9-116

**Published:** 2014-06-27

**Authors:** Hitoshi Igai, Mitsuhiro Kamiyoshihara, Natsuko Kawatani, Takashi Ibe, Kimihiro Shimizu

**Affiliations:** 1 Department of General Thoracic Surgery, Maebashi Red Cross Hospital, 3-21-36 Asahi-cho, 371-0014, Maebashi, Gunma, Japan

**Keywords:** Sternum, Intraosseous schwannoma

## Abstract

The preoperative diagnosis of intraosseous schwannoma is challenging because of its rarity. We report a resected case of sternal intraosseous schwannnoma mimicking late recurrence of breast cancer.

A 60-year-old Japanese woman with a history of breast cancer was diagnosed as having a sternal tumor by chest computed tomography (CT) demonstrating a round, well-defined, low-density nodule measuring 3.3 × 2.8 cm, which was located almost at the center of the sternum and associated with bone lysis and erosion. [^18^ F]Fluorodeoxyglucose positron emission tomography (FDG-PET)/CT demonstrated FDG accumulation in the tumor, suggesting malignancy. Therefore, late isolated recurrence of breast cancer was suspected. Surgical resection was performed for both confirmation of the diagnosis and treatment.

Pathological examination revealed that the tumor was composed predominantly of spindle-shaped cells arranged in a typical palisading pattern, being compatible with schwannoma. Although the periosteum was intact, the tumor was found to have destroyed the cortex of the sternum and proceeded forward to the bone marrow. Additionally, immunohistochemical staining revealed that the lesion was diffusely and strongly positive for S-100 protein. Thus metastasis from breast cancer was ruled out on the basis of the features revealed by microscopy.

## Background

Among primary bone tumors, intraosseous schwannoma accounts for less than 0.2% [1]. These tumors typically develop in the mandible, sacrum, maxilla, fibula, ulna, vertebrae, and other bones. The sternum is a considerably rare site for intraosseous schwannoma, and only two such cases have been reported [2,3].

The preoperative diagnosis of intraosseous schwannoma is challenging because of its rarity and the fact that it is not readily recognized by physicians. Herein, we reported a resected case of sternal intraosseous schwannnoma mimicking late recurrence of breast cancer.

## Case presentation

**Figure 1:**
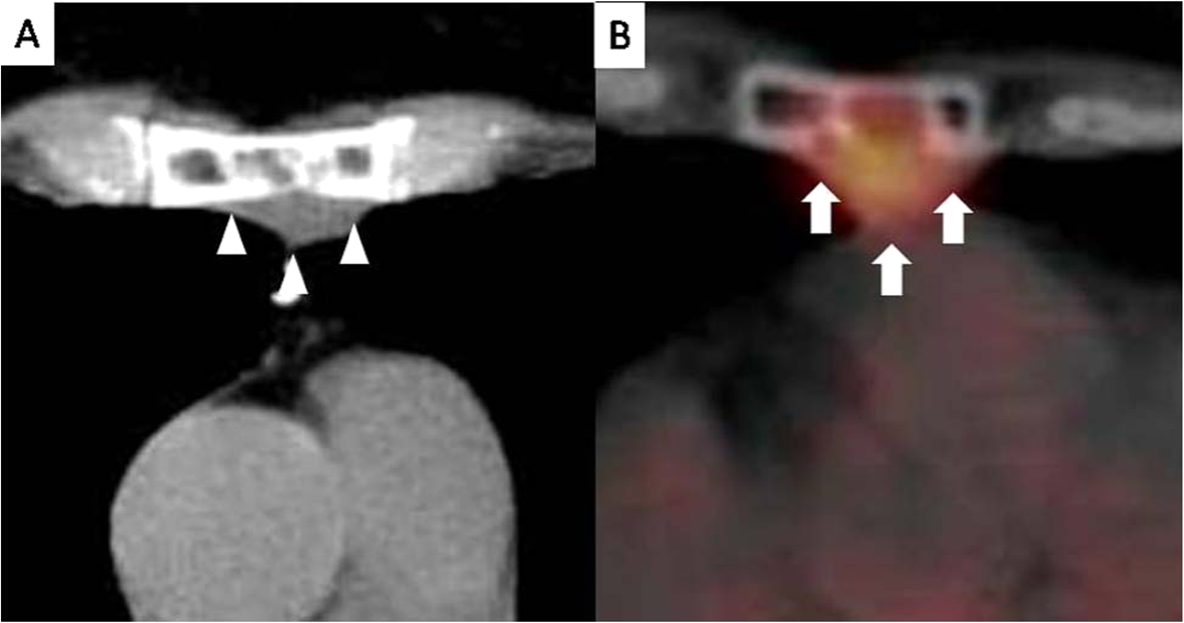
**The finding of chest CT. (A)** Chest computed tomography showed a round, well-defined, low-density nodule measuring 3.3 x 2.8 cm (arrows), which was located at almost the center of the sternum with bone lysis and erosion. The finding of FDG-PET/CT **(B)** [^18^ F]Fluorodeoxyglucose positron emission tomography/computed tomography demonstrated fluorodeoxyglucose accumulation (arrowheads) in the tumor. The standardized uptake value was 5.51.p.

A 60-year-old Japanese woman consulted our hospital complaining of narrowing of the visual field. Chest computed tomography (CT) for screening demonstrated a round, well-defined, low-density nodule measuring 3.3 × 2.8 cm, which was located almost at the center of the sternum and associated with bone lysis and erosion (Figure 1-A). [^18^ F]Fluorodeoxyglucose positron emission tomography (FDG-PET)/CT demonstrated FDG accumulation in the tumor, suggesting malignancy (Figure 1-B). The maximum standardized uptake value (SUV) of the tumor was 5.51. As the patient had undergone resection of breast cancer 20 years previously, we suspected that the sternal tumor was a late isolated recurrence of the breast cancer.

**Figure 2:**
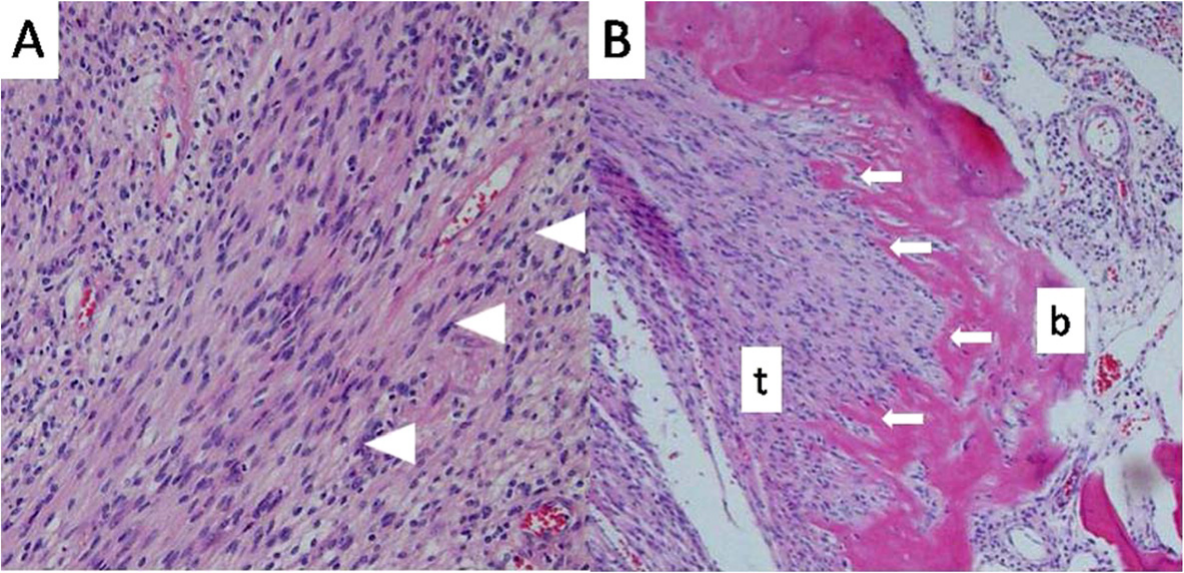
**Microscopic findings of the tumor-1. (A)** The tumor was composed predominantly of spindle-shaped cells (arrowheads) arranged in a typical palisading pattern that was compatible with schwannoma (hematoxylin and eosin; x100). Microscopic findings of the tumor-2 **(B)** The tumor (t) was found to have destroyed the cortex of the sternum (arrows) and proceeded forward to the bone marrow (b) (hematoxylin and eosin; x40).

Surgical resection was planned for confirmation of the diagnosis and treatment. Intraoperatively, a solid and round tumor was found at the back of the patient’s sternum, and was strongly attached to it. Partial resection of the sternum at the 3rd to 5th rib level was performed, followed by chest wall reconstruction using a titanium plate covered with Marlex mesh (Chevron Phillips Chemical Company LP, The Woodlands, Tx, US).Pathological examination revealed that the tumor was not encapsulated. It was composed predominantly of spindle-shaped cells arranged in a typical palisading pattern, being compatible with schwannoma (Figure 2-A). Although the periosteum was intact, the tumor was found to have destroyed the cortex of the sternum and proceeded forward to the bone marrow (Figure 2-B). Additionally, immunohistochemical staining revealed that the lesion was diffusely and strongly positive for S-100 protein. Thus metastasis from breast cancer was ruled out on the basis of the features revealed by microscopy.

## Discussion

It is difficult to diagnose sternal intraosseous schwannoma preoperatively because of its rarity. Mizuno et al. suggested some characteristic CT features of sternal intraosseous schwannoma [2], including the presence of a poorly enhanced soft tissue mass accompanied by bone lysis or erosion which were correlative with pathologically destroying the cortex of the sternum. Partial defects of the bone cortex may also be evident. These features were evident in the present case. However, findings indicative of invasiveness are also present in malignant disease. Beaulieu et al. reported that FDG uptake by schwannoma can be variable [4]. In the nine cases they reported, the SUVmax of the tumor varied between 1.9 and 7.2. Thus, it is concluded that FDG-PET/CT has limited value for differentiating benign schwannoma from malignant disease, as our case.

Although a long-term follow-up has not been accomplished in our case, it is commonly believed that the prognosis of the patient with intraosseous schwannnoma is favorable after a complete resection [2].

The features of the present case were strongly suggestive of late recurrence of breast cancer, because sometimes breast cancer is known to show late recurrence (10 to 20 years after initial treatment), and isolated recurrence at the chest wall is comparatively common [5].

## Conclusion

We have described a rare resected case of sternal intraosseous schwannoma mimicking breast metastasis, which to our knowledge is the first of its kind to be reported in the English literature.

## Consent

Written informed consent was obtained from the patient for publication of this Case report and any accompanying images. A copy of the written consent is available for review by the Editor-in-Chief of this journal.

## Competing interests

The authors declare that they have no competing interests.

## Authors’ contributions

IH is corresponding author, carried out revision of the manuscript. KM carried out revision of the manuscript. KN carried out the review of the medical record. IT carried out the review of the medical record. SK carried out revision of the manuscript. All authors read and approved the final manuscript.
